# The Use of Tissue Engineering to Fabricate Perfusable 3D Brain Microvessels *in vitro*

**DOI:** 10.3389/fphys.2021.715431

**Published:** 2021-08-31

**Authors:** Kalpani N. Udeni Galpayage Dona, Jonathan Franklin Hale, Tobi Salako, Akanksha Anandanatarajan, Kiet A. Tran, Brandon J. DeOre, Peter Adam Galie, Servio Heybert Ramirez, Allison Michelle Andrews

**Affiliations:** ^1^Department of Pathology and Laboratory Medicine, Lewis Katz School of Medicine at Temple University, Philadelphia, PA, United States; ^2^Department of Biomedical Engineering, Rowan University, Glassboro, NJ, United States; ^3^The Center for Substance Abuse Research, Lewis Katz School of Medicine at Temple University, Philadelphia, PA, United States; ^4^Shriners Hospitals Pediatric Research Center, Philadelphia, PA, United States

**Keywords:** BBB, NVU, microfluidics, bioengineering, brain endothelial cells

## Abstract

Tissue engineering of the blood-brain barrier (BBB) *in vitro* has been rapidly expanding to address the challenges of mimicking the native structure and function of the BBB. Most of these models utilize 2D conventional microfluidic techniques. However, 3D microvascular models offer the potential to more closely recapitulate the cytoarchitecture and multicellular arrangement of *in vivo* microvasculature, and also can recreate branching and network topologies of the vascular bed. In this perspective, we discuss current 3D brain microvessel modeling techniques including templating, printing, and self-assembling capillary networks. Furthermore, we address the use of biological matrices and fluid dynamics. Finally, key challenges are identified along with future directions that will improve development of next generation of brain microvasculature models.

## Introduction

The blood-brain barrier (BBB) is a highly organized and dynamic structure that acts as a physiological barrier between the central nervous system (CNS) and cerebral blood flow. It continuously regulates the passage of molecules and ions at the capillary level to maintain brain homeostasis (Abbott, 2013). Specialized brain microvascular endothelial cells (BMVECs) with distinct barrier functionalities distinguish the brain capillaries from other peripheral vessels due to the presence of highly developed and organized tight junctional complexes (TJCs) as well as molecular transporters (Bazzoni and Dejana, 2004; Saili et al., 2017; Castro Dias et al., 2019). The cell-cell interaction of BMVECs influences most molecular traffic to take a transcellular route, from the luminal (apical) to the abluminal (basolateral) side of ECs, which provides selectivity for the barrier (Abbott, 2013; Lochhead et al., 2020). Additionally, the increased contact points of TJs restrict movement in the plasma membrane from the apical and basolateral side, contributing to a polarized expression of proteins (Bazzoni and Dejana, 2004). Entry for molecules, like glucose, pass through GLUT1 transporters while large hydrophilic molecules like peptides and proteins are generally restricted, unless shuttled through receptor-mediated transcytosis (Bickel et al., 2001). BMVECs also express multiple efflux pumps on their luminal surface that regulate uptake of larger lipophilic molecules, including many drugs, into the brain (Abbott et al., 2010). Conversely, blood gases like CO2 and O2, diffuse freely across the lipid membrane down their concentration gradients.

In addition to the BMVECs, neurons, glial cells, pericytes, and the astrocytic end-feet that surround vessels form a functional unit (Abbott et al., 2006). Pericytes embedded in the basement membrane wrap around the endothelium and contribute to astrocytic polarization (Bonkowski et al., 2011; Gundersen et al., 2014). The BBB is not a standalone structure; supporting cells play a significant role in the signaling of BBB modalities to regulate the cerebral microenvironment under dynamic physiological or pathological conditions. Together, the association of these components form what is known as the “neurovascular unit” (NVU) (Zhang et al., 2012).

For decades, animal and 2D models have advanced our understanding of BBB biology. While informative, *in vivo* studies are resource and time consuming, and the species differences between humans and animal have minimized the translatability of the results to clinical applications. A lack of predictive outcomes is attributed in part due to diverging features like cell-cell signaling, transporter expression, and other physiological differences (Aday et al., 2016; Song et al., 2020). *In vitro* models, offer the ability to use human primary cells, which can bridge the gap to clinical translation by minimizing species differences. Additionally, *in vitro* models are tunable and provide a platform to finely control various aspect of BBB properties. However, planar *in vitro* models fail to mimic the complex features of the NVU. The most commonly used Boyden chamber system, consisting of a porous membrane separating two compartments, fails to facilitate the cylindrical geometry of *in vivo* vasculature as well as dynamic mechanical stimuli exerted by fluid flow. Shear stress and cyclic strain caused by physiological fluid flow have been shown to be critical to microvascular function and high *trans-*endothelial electric resistance (TEER) values (Colgan et al., 2007; Cucullo et al., 2013). Furthermore, many organ-on-a-chip platforms are based on 2D models of the BBB that cannot replicate the complex network topology of vessels formed *in vivo*. Thus, there is a pressing need to develop a functional *in vitro* model that recapitulates the key relationships between cells and the dynamic nature of the NVU.

Recent advances in biomaterials and microfluidic device fabrication allows for the recreation of a more physiologically relevant model. In this prospective, we primarily focus on the various approaches to create 3D BBB tubular model designs (vessel formation, structural support, physical characteristics), then evaluate future directions and challenges. A word about terminology related to the use of the word microvessels. In its broadest definition, the sizes of blood vessels in the human body range from capillaries (<8 μm), microvessels (<1 mm), small vessels (1–6 mm), and large vessels (>6 mm in diameter) (Chang and Niklason, 2017; Zhou et al., 2020). Cross-sectional measurements of brain microvessels derived from casting studies of human brains shows arterioles and arteries in the range of 50–240 μm while post-capillary venules and veins range from 20 to 380 μm (Duvernoy et al., 1981; Reina-De La Torre et al., 1998). A large majority of the vasculature in the brain is capillary which are found to range from 3 to 7.5 μm (Duvernoy et al., 1981; Reina-De La Torre et al., 1998). Therefore, in this perspective the use of the term microvessel, refers to vascular structures that are sized below small vessels (under 1 mm). We do not discuss *in vitro* organoids, another emerging area, due to the difficulty associated with applying fluid flow through microvessels at controlled and consistent flow rates. Overall, the next generation of 3D models has great promise to advance discoveries and treatments in areas of toxicology, drug delivery, neuropathology, infectious agents, and gene therapy.

## 3D Brain Microvessel Fabrication

**FIGURE 1:**
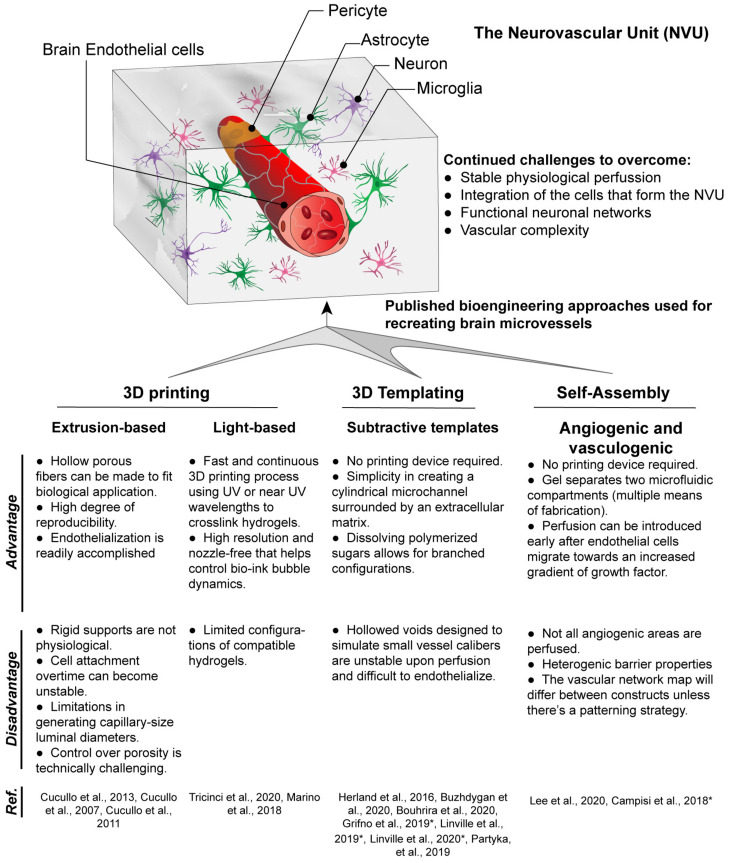
Techniques for fabrication of 3D brain microvessels and capillary-like structures. Techniques that have been used to create 3D BBB structures include 3D printing, 3D templating, and self-assembled microvascular networks. Advantages and disadvantages for each are presented as well as references for each technique used to model the BBB. (*) Indicates models that use iPSC-derived ECs which are brain endothelial-like, as recent RNA-Sec data suggests these are more neuro-epithelial (Lu et al., 2021).

Multiple vascularization techniques have been developed to create 3D brain microvessel models *in vitro*, including 3D templating technique, 3D printing, and self-assembling-based techniques (Figure 1), which will be discussed below in detail.

### 3D Templating

The most commonly used 3D microvessel modeling technique for the BBB is the 3D templating technique (Partyka et al., 2017; Grifno et al., 2019; Linville et al., 2019, 2020b,c; Bouhrira et al., 2020; Buzhdygan et al., 2020). This method creates a cylindrical microchannel surrounded by extracellular matrix (ECM), which can be lined with endothelial cells (ECs). The cylindrical microchannel is formed by inserting a cylindrical object (e.g., microneedle, thin rod, wire) into an ECM (hydrogel), typically collagen type I or fibrin, and removing the cylindrical object after the ECM is fully formed. An alternative methodology to accomplish a similar cylindrical microchannel is through Saffman–Taylor instability, in which a low viscosity liquid displaces a more viscous fluid from an ECM thus forming the cylindrical vessel (Herland et al., 2016). The 3D templating technique is only suitable to construct large linear blood vessels ranging from 60 to 700 μm (Price et al., 2010; Bogorad et al., 2015; Wang et al., 2018). Then ECs are introduced into the inner channel to form a monolayer that lines the lumen. However, smaller vessel diameters increase the difficulties in sufficiently seeding EC to create a fully confluent vessel, though a recent study demonstrated the potential of using growth factor gradients to aid in endothelialization (Linville et al., 2016). Perhaps more crucially, this 3D templating technique is incapable of yielding the branched networks due to the manner in which the cylindrical object is removed from the ECM (Price et al., 2010; Buchanan et al., 2014). However, simplistic bifurcation models are possible such as that by Bouhrira et al. (2020). Although not a true branched vasculature, the sharp turn in fluid entry creates disturbed flow patterns in both steady and physiological waveforms (Bouhrira et al., 2020, 2021). Most 3D microvessel models consist of a single vessel, however, multiple side by side vessel scaffolds can also be created (Linville et al., 2020a). The dual vessel design can be used for access to the luminal vs. abluminal compartment. This configuration can allow for the generation of hydrostatic pressure and mechanical stress as well as to measure transendothelial-electrical resistance (TEER) (Partyka et al., 2017).

### 3D Printing

In contrast to the templating technique, 3D printing employs principles of additive layer-by-layer deposition and sacrificial removal of filler material. Additionally, living cells can be printed in a 3D space using computer-aided design. There are two major technologies that have been used for the BBB: extrusion- and light-based bioprinting.

In the *Extrusion-based printing (EBB) technique*, biomaterials are extruded out of the printhead or nozzle by applying mechanical or pneumatic pressure. Inorganic substates are commonly extruded to create thin tubes which serve as a scaffold for a model of the BBB (Cucullo et al., 2007, 2011, 2013). Although some materials naturally create a porous structure during the extrusion process, most require additional micromachining to create uniform pores (Cucullo et al., 2011). Limitations include a thick wall which prevents the interactions of cells grown on the inner lumen and those on the outside surface and the size of extruded structures is limited by the nozzle size. For example, the dynamic *in vitro* blood-brain barrier (DIV-BBB) utilizes hollow polypropylene fibers which are 150 μm thick and prevents any direct cell interactions (Cucullo et al., 2007, 2013). Many inorganic scaffolds are not translucent and thus visualizing the cells inside to verify a completely endothelialized structure is more challenging. Another aspect of inorganic substrates is that they are generally more rigid than organic ones. These inorganic scaffolds have not been used with a surrounding matrix but future models could incorporate a hydrogel to generate a parenchymal microenvironment. In addition to indirect printing of scaffolding templates, direct printing by extrusion is an alternative method which allows the incorporation of cells and biomolecules but has yet to be used in models of the BBB.

**FIGURE 2:**
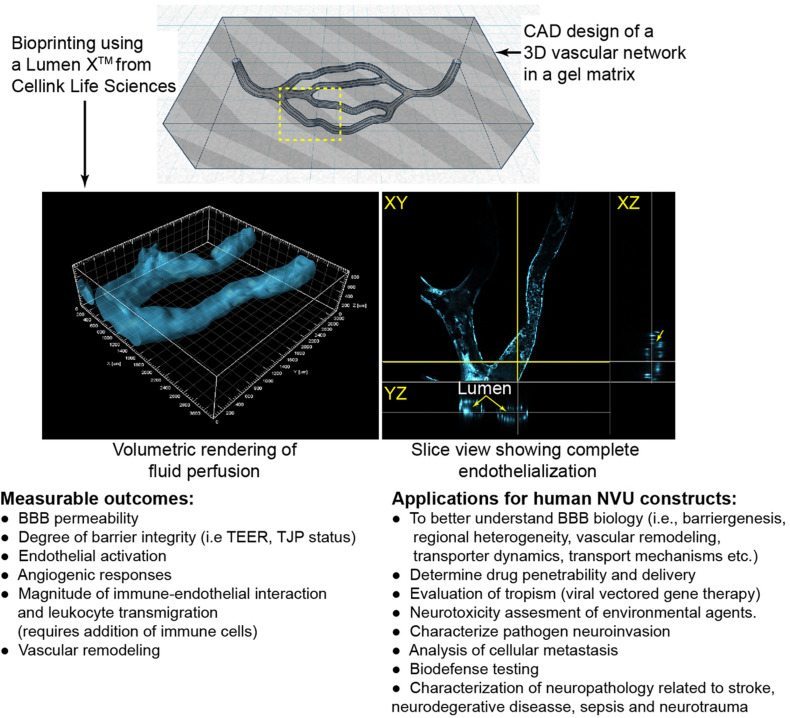
Example of light-assistant bioprinting of 3D constructs and endothelialization with primary human brain microvascular endothelial cells (hBMVECs). 3D vascular scaffold was created using computer aided design (CAD) software and then were printed of PEGDA-GelMA using the LumenX^TM^. hBMVECs were grown under perfusion until completely confluent. Vessels ranged from 225 to 400 μm in diameter. Possible applications and measurable outcomes for 3D constructs are described.

*Light-based printing (LBB)* is a very fast and continuous 3D printing process which uses light as the energy source to crosslink biomaterials to form a scaffold. Most commonly utilized is UV or near UV wavelengths to polymerize hydrogels which may contain cells and proteins. This technique offers several advantages including very high resolution, creation of complex branching and tapering of vessel scaffolds in a 3D space (Grigoryan et al., 2019). With regards to BBB models, two-photon lithography has recently been employed to construct a series of tubes with an average diameter of 10 and 2 μm wall thickness (Marino et al., 2018; Tricinci et al., 2020), close to capillary size. These structures resemble capillaries in size with the ability of a single endothelial cell to construct the lumen and for interactions with cells grown on the outer surface. As with the studies involving extruded tubes, these capillary tubes are not translucent and have not been used with a 3D matrix around the vessels. In addition to the fine scale resolution of two-photon lithography utilized by Marino et al. (2018) and Tricinci et al. (2020), LBB has promising capabilities for branched 3D BBB models. Using the Lumen X^TM^, we demonstrate the ability to create scaffolds with a branching network. Furthermore, primary human brain microvascular endothelial cells completely line the scaffolding structure forming a lumen and the vessels transverse the matrix in the *Z*-axis (Figure 2).

### Self-Assembled Microvasculature

Self-assembled-based techniques involve cells spontaneously organizing into capillary-like structures with the key presence of a lumen. These techniques can be split between angiogenic and vasculogenic approaches. Angiogenic strategies are based on cells invading into the surrounding hydrogel, whereby groups of ECs sprout, migrate, and organize to form new tubular configurations eventually forming blood vessels (Vailhé et al., 2001). The random sprouting through angiogenesis is more similar to *in vivo* biological processes. However, a limitation associated with angiogenesis is the replicability between constructs. Vessels sprout spontaneously, in different ways every time and some create overly dense networks (Haring and Johnson, 2020). The formation of these vessels is best with the structural support of fibroblasts or pericytes, which makes them a natural model for recreating the BBB (Campisi et al., 2018; Lee et al., 2020). Additionally, most angiogenic-based models have been used only with interstitial flow and higher capillary level shear stresses are difficult to achieve due to the angle of angiogenic spouts from the main vessels. Vasculogenic approaches involve culturing endothelial cells and pericytes within a hydrogel and allowing for the formation of microvessels over time. This strategy has been used to create capillary scale BBB-forming microvessels within collagen/hyaluronan composite hydrogels (Partyka et al., 2019; Tran et al., 2020). Similar to the angiogenic method, the vasculogenic protocol prohibits patterning of complex topologies since the structures are formed spontaneously. However, the application of interstitial flow can align these microvessels in a manner similar to non-BBB microvessels (Morin et al., 2014).

## Biological Matrices to Support Perfusable Microvessels

The fabricating materials used to design the microvessel must be biocompatible. In 3D bioprinting, the bio-inks must retain the biological, physical and mechanical requirements during the operation of printing. Bio-inks with or without living cells are commonly used to configure scaffolds with microvessel structures. Generally, these crosslinkable substances include hydrogels such as collagen, gelatin, fibrin, alginate, chitosan, poly (ethylene glycol)-PEG hydrogels, poly(2-hydroxyethyl methacrylate)-pHEMA and poloxamers (Charnley et al., 2009; Wüst et al., 2011; Billiet et al., 2012; Vasile et al., 2020). These hydrogels have different properties for cell culture and growth. Some bio-inks are available in varying molecular weights which can impact the stiffness of the surrounding ECM. While stiffer substrates can be easier to manipulate, substrate stiffness and matrix density can affect EC spreading in some cases preventing full formation of endothelial cell-cell connections. Although, combining collagen hydrogels and polymer crosslinkers can improve cell attachment and the density of the collagen can encourage optimal endothelial sprouting (Crosby and Zoldan, 2019). It may also be necessary to include additional ECM components such as fibronectin, laminin, poly-lysine and large glycosaminoglycans (i.e., hyaluronic acid) to reinforce cell adhesion. Bio-inks are commonly mixed with living cells prior to the crosslinking process thus creating a scaffolding structure with cells embedded in the matrix.

## Flow Dynamics and Stability of Microvessels

The cerebral vascular system exhibits different shear stress levels depending on the rate and velocity of blood flow. The typical physiological shear stress level of healthy arteries range from 10 to 70 dynes/cm (Saili et al., 2017), whereas healthy veins range from 1 to 6 dynes/cm^2^ (Fung, 1997; Malek et al., 1999; Paszkowiak and Dardik, 2003). In brain capillaries, the blood flow is mostly heterogenous and have a broad range of shear stress levels due to the variation of vessel diameters (Mairey et al., 2006).

In particular, hemodynamic shear stress is known to modify morphology differentiation, reorganization, alignment and remodeling behaviors of EC that are significant for microvessel formation. Higher shear stress promotes EC survival and cause cells to align parallel with the direction of flow, while lower shear stress levels enhances EC apoptosis, proliferation, permeability, and shape change leading to vasoconstriction, coagulation, and platelet aggregation (Krizanac-Bengez et al., 2004). The shear stress, τ, applied to ECs by laminar flow of culture medium, which is a Newtonian fluid, can be calculated using the Poiseuille equation (Lipowsky, 1995; Fung, 1997; Malek et al., 1999; Paszkowiak and Dardik, 2003; Galpayage Dona et al., 2020a).

$$\tau =\frac{4\,\mu \,Q}{\pi \,r^{3}}$$

where μ is the viscosity of the blood, (μ∼ 0.0035 Pa.s), *Q* is the blood flow rate and *r* is the vessel radius.

Shear stress is a critical physiological parameter, however, perfusing 3D gels poses unique challenges relate to the mechanical properties (i.e., stiffness, stress relaxation, degradation, self-healing). It is important to note that few 3D brain microvessel models support physiological shear stresses (Cucullo et al., 2007, 2011, 2013). This may be in part due to the lack of matrices able to support the higher flow rates and the difficulties in obtaining a leak-free closed system. For that purpose, the future of 3D printed microvascular structure must address the necessity of higher shear stress level and mechanical properties of gel materials.

Studies on peripheral ECs have used different fluid flow patterns such as laminar, pulsatile, and turbulent flow to regulate shear stress of the 3D printed complex microstructure of a porous scaffold to employ well-formed EC monolayer (Song et al., 2005; Shao et al., 2009; Cui et al., 2019; Kinstlinger et al., 2020). These types of *in vitro* microfluidic studies are lacking in the field of the cerebral vasculature biology. However, Bouhrira et al. (2021) designed a flow system capable of generating complex, physiologically relevant flow profiles in a linear 3D BBB model. Branched networks and varying diameters adds a layer of intricacy to the shear stresses in vasculature structures. For instance, Kinstlinger et al. (2020) studied the flow rate of different dendritic vascular networks and observed a significant deviation between theoretical and experimental flow rate and shear stress values. These changes were explained by the fact of a small deviation of vascular radius through the network lead to a significant change of shear stress value (Eq. 1) (Kinstlinger et al., 2020). In regard to self-assembled networks, shear stress offered a tunable parameter for controlling bifurcations and branching. Perhaps appliable to brain ECs, Ueda et al. (2004) showed in Bovine pulmonary microvascular endothelial cells that the number of bifurcations and endpoints increased for networks exposed to shear stress, whereas the number of bifurcations alone increased for networks not exposed to shear stress. Moreover, the stability of the vessel can be achieved by addition of drainage channels or tapering the vessels (Khan and Sefton, 2011), by controlling the pressure of the vessel along the length of it.

Important to consider that *in vivo*, ECs experience the mechanical forces from the fluidic movement and interactions with blood components. Blood, in contrast to culture medium, is non-Newtonian and its boundary layer is affected by the thickness of the cell free layer, a phenomenon caused by red blood cell streaming (shear thinning). Multiple factors including hematocrit and vessel diameter, a relationship referred to as the Fahraeus-Lindquist effect, alter the magnitude and dynamics of shear stress applied to the endothelium. Additionally, the glycocalyx contributes to the homogenous blood flow distribution and mediating interactions between red blood cells with the microvasculature wall (Secomb et al., 1998; McClatchey et al., 2016). Therefore, incorporation of blood is necessary to gain a better understanding of how shear stress affects barrier structure and function.

## Perspectives, Future Directions, and Conclusion

Advances in tissue engineering and microfabrication enhance our ability to create new models of the BBB that more accurately mimic the *in vivo* structure and environment. Key areas of growth for the field include capillary structures capable of maintaining physiological shear stress levels, branched and multi-caliber vessel networks in a 3D space, as well as the inclusion of functional neurons in various anatomical configurations. To date, no 3D microfluidic model of the BBB incorporates functional neurons to create a true neurovascular unit.

In corporation of analytic measurements is an area for future improvement. For instance, microfluidic based electrical impedance techniques have been widely used to characterize the barrier related parameters and cellular and physical properties of cells (Cucullo et al., 2008; Douville et al., 2010; Szulcek et al., 2014; Reiss and Wegener, 2015; Bischoff et al., 2016; Galpayage Dona et al., 2017, 2020b), but few 3D models have real-time continuous measurements (Cucullo et al., 2007). Instead most rely on electrodes inserted into ports connected to the inner and outer luminal compartment requiring cessation of perfusion to obtained single timepoint measurements. New innovative designs that incorporate micro electrodes into these 3D spaces could yield outputs in real-time. Additional measurable parameters could include evaluations of metabolites, nitric oxide etc.

For the formation of new 3D scaffolding, one potential area of exploration includes the use of degradable materials (Qiu et al., 2019). This methodology has the potential to create a solid foundation for brain microvessels that afterward can be dissolved leaving the vessel behind. Also, 3D bioprinters have the capability to meet the demands of creating novel 3D branched and multi-caliber vessel networks. Although 3D bio-printers have high precision and reproducibility, the majority of printable networks have not reached capillary size yet. The developmental of new bioinks and devices with greater resolution can overcome existing hurdles.

Another primary challenge facing the field is the selection of an appropriate cell source to mimic brain endothelium. A recent study used RNA-sequencing demonstrated that iPS-derived cells used for BBB models are more epithelial in nature, which explains their ability to exhibit high TEER values (Lu et al., 2021). Therefore, a better understanding of the differentiation programming of iPS-derived cells is needed to produce a brain endothelial phenotype. Another alternative to immortalized cell lines such as hCMEC/D3 is primary cells, though these sources are scarce. Primary adult human brain endothelial cells are in short supply commercially and healthy tissue derived from resections in surgical treatment of epilepsy are becoming less common (Bernas et al., 2010). Conversely, human brain endothelial cells isolated from fetal tissue have properties similar to adult primary cells and can be obtained from a wider range of donors and sexes (Andrews et al., 2018). An additional advantage from the use of human fetal tissue is the ability to isolate multiple cells of the neurovascular unit from the same donor (Andrews et al., 2018).

In summary, 3D tissue engineered models of the NVU requires the intersection of multiple fields of study, such as engineering, material science, cell biology, microfabrication and specialized expertise depending on application (toxicology, microbiology, neuroscience etc.). Undoubtedly, the advent of the next generation of physiologically akin NVU microfluidic models will greatly advance our ability to provide needed solutions for neurological diseases and disorders.

## Data Availability Statement

The raw data supporting the conclusions of this article will be made available by the authors, without undue reservation.

## Author Contributions

AMA, SR, and PG contributed to the conception, outline, and editing of this perspective. BD and KT edited the manuscript. TS and AA wrote sections of the manuscript. KG generated the construct shown in Figure 2 and wrote sections of the manuscript. JH performed the imaging and image analysis shown. All authors contributed to manuscript revision, read, and approved the submitted version.

## Conflict of Interest

The authors declare that the research was conducted in the absence of any commercial or financial relationships that could be construed as a potential conflict of interest.

## Publisher’s Note

All claims expressed in this article are solely those of the authors and do not necessarily represent those of their affiliated organizations, or those of the publisher, the editors and the reviewers. Any product that may be evaluated in this article, or claim that may be made by its manufacturer, is not guaranteed or endorsed by the publisher.
